# “I can’t describe how I could get better, but I would like to” - Conception of health and illness of refugee youth in Germany

**DOI:** 10.3389/fpsyg.2023.1107889

**Published:** 2023-05-12

**Authors:** Anna Swantje van der Meer, Friederike Durlach, Katharina Szota, Hanna Christiansen

**Affiliations:** Department of Child and Adolescent Psychology, Philipps-University of Marburg, Marburg, Germany

**Keywords:** children and adolescents, mental health literacy, qualitative research, psychotherapy, refugee, refugee mental health

## Abstract

**Introduction and objective:**

Almost half of all the people displaced worldwide are children and adolescents. Many refugee children, adolescents, and young adults suffer from psychological stress. However, their utilization of (mental) health services is low, probably due to a lack of knowledge about (mental) health and (mental) health care. The current study aimed to explore concepts of (mental) health and illness of refugee youth as well as assess their mental health literacy (MHL) to arrive at conclusions for improving mental health care access and use.

**Method:**

From April 2019 to October 2020, we conducted 24 face-to-face interviews with refugee children and adolescents in an outpatient clinic (*n* = 8), in youth welfare facilities (*n* = 10), and at a middle school (*n* = 6). A semi-structured interview was used to assess knowledge about mental and somatic health and illness as well as corresponding health strategies and care options. The material was evaluated using qualitative content analysis.

**Results:**

Participants (*N* = 24) were between 11 and 21 years old (*M* = 17.9, *SD* = 2.4). The coded material was assigned to four thematic main areas: (1) conception of illness, (2) conception of health, (3) knowledge about health care structures in their country of origin, and (4) perceptions of mental health care structures in Germany. Compared to somatic health, the interviewed refugee children and adolescents knew little about mental health. Furthermore, respondents were more aware of opportunities of somatic health promotion, but almost none knew how to promote their mental health. In our group-comparative analysis we observed that younger children possess little knowledge about mental health-related topics.

**Conclusion:**

Our results show that refugee youth have more knowledge about somatic health and somatic health care than about mental health (care). Accordingly, interventions to promote the MHL of refugee youth are necessary to improve their utilization of mental health services and to provide adequate mental health care.

## Introduction

Existing, resurgent, and escalating armed conflicts, as well as the consequences of global warming as results of climate change, are creating continuous dynamic refugee movements worldwide. By October 2022, the United Nations High Commissioner for Refugees [United Nations High Commissioner for Refugees (UNHCR), 2022] estimates that around 14.6 million people have crossed the Ukrainian border because of attacks by the Russian military in the country. The number of internally displaced people (IDP) in Afghanistan rose for the 15th year in a row. Also due to the Taliban’s return to power in August 2021, 900,000 people were displaced within that country or to neighboring countries. The Internal Displacement Monitoring Center assumes that around 23.7 million people were displaced within their own countries because of extreme weather occurrences like storms, droughts, and floods in 2021 [United Nations High Commissioner for Refugees (UNHCR), 2022].

At the end of 2012, 42.7 million people were forcibly displaced. By the end of 2021, the number of people forcibly displaced had already grown worldwide to 89.3 million [United Nations High Commissioner for Refugees (UNHCR), 2022]. More than 69% of the refugee population originated from the Syrian Arab Republic (6.8 million), Venezuela (4.6 million), Afghanistan (2.7 million), South Sudan (2.4 million), and Myanmar (1.1 million). The largest number of hosted refugees is in Turkey, reported to be 3.8 million, followed by Colombia hosting over 1.8 million people. Germany hosted the fifth largest number of people with almost 1.25 million people, with Syrian refugees constituting the largest group [United Nations High Commissioner for Refugees (UNHCR), 2022].

Between 2018 and 2021, 1.5 million children were born as refugees [United Nations High Commissioner for Refugees (UNHCR), 2022]. Furthermore, over 41% of the forcibly displaced people worldwide were minors. From January to September 2022, a total of 56,562 children and adolescents under the age of 18 applied for asylum in Germany according to the Federal Office for Migration and Refugees [Bundesamt für Migration und Flüchtlinge (BAMF), 2022]. In 2021, nearly half (41.9%) of 148,200 asylum seekers were minors according to the Federal Office for [Bundesamt für Migration und Flüchtlinge (BAMF), 2022]; [United Nations High Commissioner for Refugees (UNHCR), 2022]. Among these, 3,249 asylum seekers were unaccompanied and separated children (UASC) according to the Association for Unaccompanied Refugee [Bundesfachverband unbegleitete minderjährige Flüchtlinge (BumF), 2021].

An unaccompanied child is a child or adolescent separated from both parents and other relatives not being cared for by any other adult, by law or custom. A separated child is a child or adolescent disconnected from both parents or from his/her previous legal or customary primary care-giver, but not necessarily from other relatives (International Committee of the Red Cross, 2004). Most UASC living in Germany are male and originate primarily from Afghanistan and the African countries of Somalia, Guinea and Eritrea [German Youth Institute/Deutsches Jugendinstitut e.V. (DJI), 2020]. UASC are taken into care by youth services and are mainly placed in youth welfare facilities (YWF). Since 2010 there has been a sharp increase in the number of cases in this regard, with a particularly high growth dynamic observed for the period between 2014 and 2016 [Deutsches Jugendinstitut e.V. (DJI), 2020].

Refugee children and adolescents have fled war, persecution and violence with their families or by themselves. Due to post-migration stressors like discrimination, uncertain residence, lack of perspectives or psychological stress caused by migration-related traumatic life events, the psychological burden of refugees in the country of arrival is persistently high (Höhne et al., 2020). Reviews further indicate high rates of mental illness among refugee children and adolescents. Particularly high prevalence rates are reported for posttraumatic stress disorder (PTSD) (22.71%), depression (13.81%) and anxiety disorders (15.77%) (Blackmore et al., 2020). There is also evidence that prevalence rates of anxiety, depression, and PTSD in children, adolescents, and adults are significantly higher in refugee populations than those reported in non-refugee populations over the globe (Henkelmann et al., 2020).

Despite the high prevalence rates of mental illnesses in refugee populations described above, studies show lower utilization rates of mental health care system structures by refugee youths (Bean et al., 2006; Colucci et al., 2014). A Swedish long-term study reported that migrant people, irrespective of age, use mental health services less often than their Swedish peers.

One reason for refugees’ low-frequency use of the mental health care system is the fear of stigmatization (by peers and society) originating from a mental-illness diagnosis (Fazel and Betancourt, 2018). Qualitative studies report an association between mental illness and negative and stigmatizing terms such as “crazy” or “wacky” which might result in young refugees’ low use of such service (Majumder et al., 2015). Another result from a qualitative analysis addressing the perspective on mental health care is that refugee children and adolescents often do not trust mental health care professionals and feel pressured by them (Jarlby et al., 2018). Not getting adequate help for their mental health problems results in them feeling poorly understood (Jarlby et al., 2018). Other barriers to access were divergent explanatory models and culturally influenced concepts of mental health and mental health care, limited access to mental health services, and the need to work with interpreters (Colucci et al., 2015). Furthermore, poor health literacy (HL) can be a reason for low frequency use of health care structures by refugees (Colucci et al., 2015).

HL is defined by “the degree to which individuals have the capacity to obtain, process, and understand basic health information and services needed to make appropriate health decisions” (Kindig et al., 2004). Studies indicate lower HL and knowledge of illness among refugees (McKeary and Newbold, 2010; Khuu et al., 2016). This might be due to various biographical factors. Refugees are exposed to higher rates of interrupted schooling because of war or lacking educational structures. Furthermore, less host-language literacy, and resettlement into areas with few people who share cultural, or religious backgrounds, lead to less social cohesion and low awareness of local health systems (McKeary and Newbold, 2010). Poor HL can lead to harmful health behaviors such as non-adherence, as well as ineffective health care system navigation, resulting in a poor health outcome even while increasingly utilizing health care structures (Fox et al., 2021).

Mental health literacy (MHL) is an extension of the HL concept and is defined as “knowledge and beliefs about mental disorders which aid their recognition, management or prevention” (Jorm et al., 1997). It includes the ability to recognize mental illness, knowing how to collect mental health information; knowledge about risk factors and causes, and about self-treatments and professional help available (Jorm, 2012). There is also evidence that different levels of knowledge and beliefs about the nature and management of mental health problems may act as barriers to help-seeking behavior in e.g., Arabic-speaking groups (Slewa-Younan et al., 2014). Following this, some MHL researchers argue that also stigma, positive mental health, and help-seeking efficacy should be added to the MHL definition (Kutcher et al., 2019; Spiker and Hammer, 2019).

Although conceptions health and illness have such a significant influence on help-seeking behavior and thus also on the health of children and young people, few studies have sought the perspectives and expectations of refugee children and adolescents on the issues of mental illness, mental health, and psychotherapy. Existing research has focused particularly on the perspective of UASC. A qualitative interview study from Denmark with ten unaccompanied refugee youths aged 17–18 years reported that respondents defined mental health as the ability to function well in and be part of a community. One possible strategy to promote this is social activities and social support (Jarlby et al., 2018). Furthermore, qualitative research showed that refugee adolescents and young adults were more receptive and able to engage in discourse on the subject of mental health when alternative terms such as “stress” were used. By using alternative words, the authors sought to prevent stigma-induced reactions that they feared would occur if they had used the term “mental health.” By using non-stigma related terms, perceptions and knowledge of respondents regarding influencing factors on mental health could be revealed (Filler et al., 2021). In contrast, a UK study demonstrated that also unaccompanied refugee youth showed very heterogeneous knowledge regarding mental illness and health in general. Their results indicated that some respondents revealed constructs of mental illness resembling those in the West, others cited physical causes mental illness, and others denied mental health problems (Demazure et al., 2021). To improve care, a more detailed understanding of the use of alternative support strategies of refugee children and young people is necessary. A systematic review on the psychosocial needs of refugee children and youth identified social support, security, culture and education as important factors (Nakeyar et al., 2018). Similarly, there is evidence that young refugees are more likely to seek out other support structures such as friends, religious or school-based services than mental-health care structures (De Anstiss et al., 2009; Ellis et al., 2010). Furthermore, prayer as well as sleeping, reading, talking to friends, exercising or watching television have been reported as means to alleviate levels of psychological distress (Halcón et al., 2004; Ellis et al., 2010; Jarlby et al., 2018).

Taking cues from the latest international research, our aim was to analyze the conceptions of (mental) health and (mental) illness of refugee children, adolescents, and young adults in Germany. We also analyzed differences between age groups and between refugee adolescents with and without mental disorders. Based on our findings, we will derive implications for psychotherapeutic practice and research as well as the state of MHL in refugee youth.

## Materials and methods

### Participants

Our sample is a consecutive convenience sample. Participants were recruited from a psychotherapeutic outpatient clinic in Germany which they visited for diagnostic assessment and psychotherapeutic treatment (study population 1 = S1), from a middle school (study population 2 = S2) and from YWF (study population 3 = S3). Participants from S2 fled accompanied by their families, participants from S1 and S3 fled to Germany unaccompanied. A total of 24 (*N* = 24) refugee children, adolescents and young adults participated in this study. In S1, eight (n = 8) patients, in S2, six (n = 6) pupils and in S3 ten (n = 10) adolescents and young adults attended the interview. Respondents met inclusion criteria if they were between 10 and 21 years old and had fled to Germany. Respondents were excluded if they had already completed or were currently undergoing psychological, psychiatric, or psychotherapeutic treatment. For respondents from the outpatient clinic, interviews had to be conducted during the diagnostic assessment and before the start of treatment. The characteristics of our study samples are summarized in Table 1.

**Table 1:** Study sample: demographic data (*N* = 24).

	S1 (*n* = 8)	S2 (*n* = 6)	S3 (*n* = 10)
Age in years: *M* (*SD*)	19.0 (1.0)	13.3 (1.0)	20.2 (1.5)
Gender			
M	8	3	10
W		3	
SDQ			
Normal		6	10
Borderline	5		
Clinically relevant	1		
Accommodation			
Independent	4		3
With parents		6	
Residential group	4		7
Interview length			
*M (SD)*	18:38 (05:25)	08:26 (01:18)	15:45 (03:14)
Country of origin			
Afghanistan	2		4
Somalia	2		2
Pakistan	2	1	
Iran	1		
Syria	1	3	
Eritrea		2	3
Sudan			1

### Data collection

The interviews were based on a semi-structured interview guideline and conducted in face-to-face sessions. We generated the interview guideline based on an extensive literature research and on the issue of interest to this project, to examine conceptions of health and illness of refugee youth, knowledge about personal (mental) health care strategies, knowledge about (mental) health care structures in the countries of origin and perceptions regarding psychotherapy in Germany. We posed several initial questions (full guidelines can be found in Supplementary material) to evoke concepts of health and illness, and then followed up with more specific prompts (e. g. regarding mental illness or personal attitudes of interviewees) at the discretion of the interviewers.

### Procedures

Participants were informed in advance *via* an information letter about our research objectives and handling of the collected data. If desired by the participants, they had the option of getting help from an interpreter. After clarification of open questions about the procedure, participants were asked to sign a consent form for the use of their data. If participants were younger than 18 years at the time of the interview, additional written informed consent from a parent or a guardian with custody was obtained. The interviews took place face-to-face in the participating outpatient clinic, in a middle school or in the YWF where the participating respondents resided. All interviews were recorded by audio equipment and stored securely in accordance with data protection requirements. Ethics approval was granted by the Ethics Committee of the Department of Psychology of Philipps University Marburg (approval number: 2020-05k). Afterwards, the interview atmosphere, general conditions, and special incidents during the interview were noted, according to the quality criteria of documentation of qualitative research (Kruse, 2015; Mayring, 2015). The interviews were conducted from April 2019 to October 2020. Details about the length of the interviews are also shown in Table 1.

### Analyses

The audio material was transcribed using the F4 transcription software and applying transcription rules according to Dresing and Pehl (2018). The participants were pseudonymized by assigning them a code containing information about the sample affiliation (S1, S2, S3) and a consecutive numbering (e.g., S1_R1 = Respondent 1 of S1).

The data evaluation of the transcribed material relied on the content structuring qualitative content analysis according to Kuckartz (2016) and was carried out with the help of analysis software MAXQDA, 2020. The aim of content structuring qualitative content analysis is to identify, categorize and systematically describe the material relevant to the research question with regard to the research aim. The qualitative content analysis according to Kuckartz (2016) is formed *via* a seven-step process.

**Figure 1:**
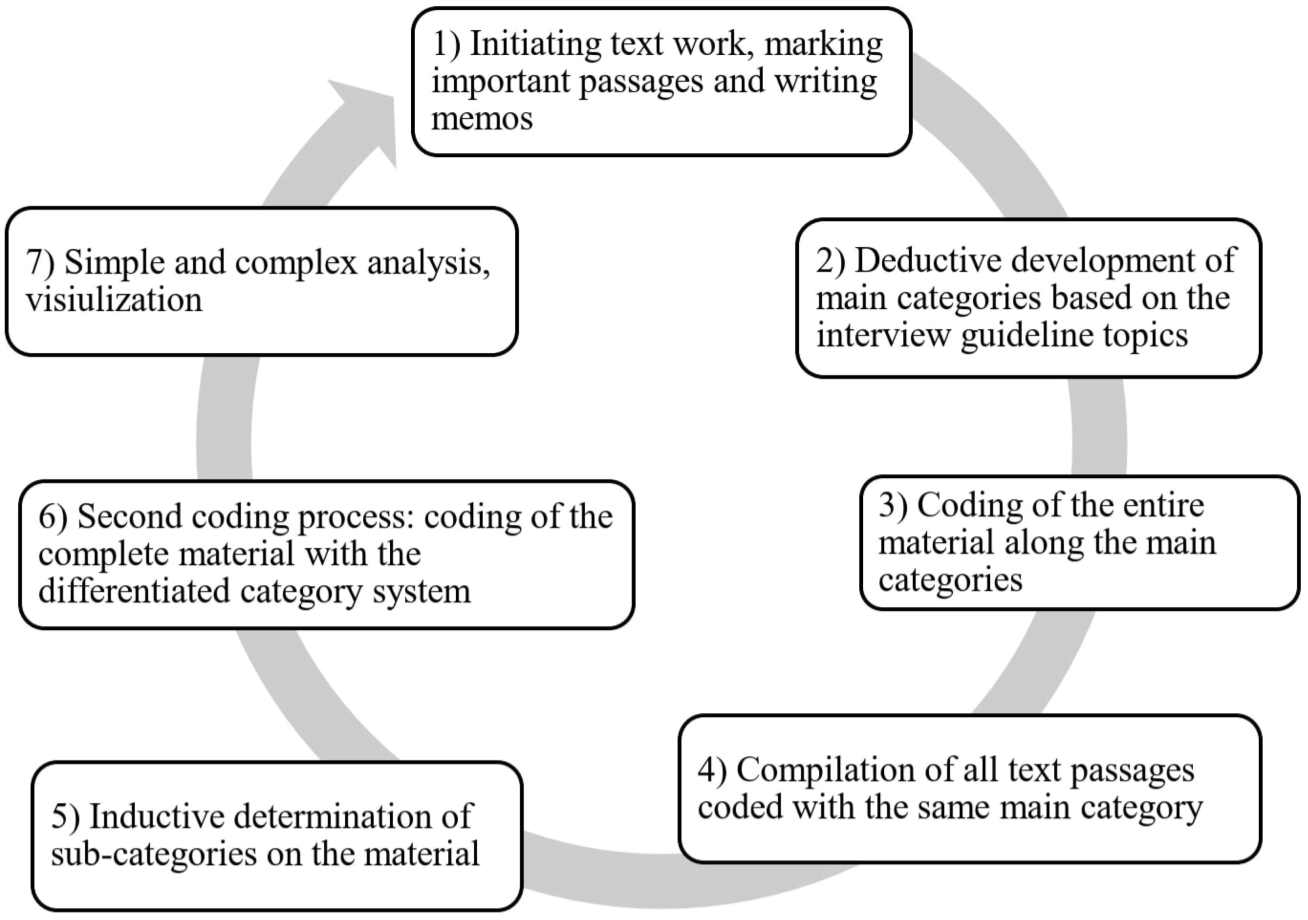
Flowchart qualitative content analysis according to Kuckartz (2016).

**Step 1**: Initiating text work, marking important text passages and writing memos. The transcribed interviews were read, relevant passages marked and comments in the form of memos on the existing content were made. To conclude the first phase of the analysis process, case summaries of each interview were prepared (Kuckartz, 2016). **Step 2**: Main categories were developed for the category system. Main categories can be built inductively (out of data) or deductively. Deductively created categories had already been created independently of the interview material, based either on the interview guidelines or a previous literature search. However, the generating of categories need not be exclusively inductive or deductive; a deductive-inductive mixed form of category formation is also possible and was applied in the present research project (Kuckartz, 2016). The possibility of a mixed form of category generating is particularly suitable here, since on the one hand, due to the structuring of the interview guideline, main and partial Sub-categories already exist before the first material pass, and on the other hand, due to the material, some Sub-categories had to be added during further passes. The category system we created was tested on the basis of four interviews (15% of the total material) and its applicability was checked by coding the material from the four interviews with the main categories already generated. The material from the four interviews could be assigned to the main categories, so it was not necessary to add new main categories to the category system. **Phase 3**: First coding process, coding of the entire material along the main categories. Corresponding text sections were assigned to the different categories. Since a text section and a single sentence can contain several topics, coding with several categories is possible and sensible (Kuckartz, 2016). After **Phase 4**: Once all text passages coded with the same category were compiled, we differentiated the categories further in **Phase 5**: Inductive determination of subcategories on the material. For this purpose, a tabular overview of the main categories with the respective Sub-categories was created and the assigned material examined. Missing subcategories were added. **Phase 6**: Second coding process: the complete material with the differentiated category system was coded. The assignment of the text passages was checked again and previously unassigned material then assigned to the newly added subcategories. **Phase 7**: Category-based evaluation and presentation of results, an attempt was made to use the category system to seek patterns in the collected material providing information about the field under investigation concerning the research questions (Kuckartz, 2016). In the present work, the material was evaluated using the category-based evaluation of the main categories. In doing so, the composition of the main category was presented according to its subcategories. For this purpose, we did not only mention frequencies, but also arrived at results in terms of content and presented those based on quotations from the texts (see Figure 1).

### Quality criteria

**Figure 2:**
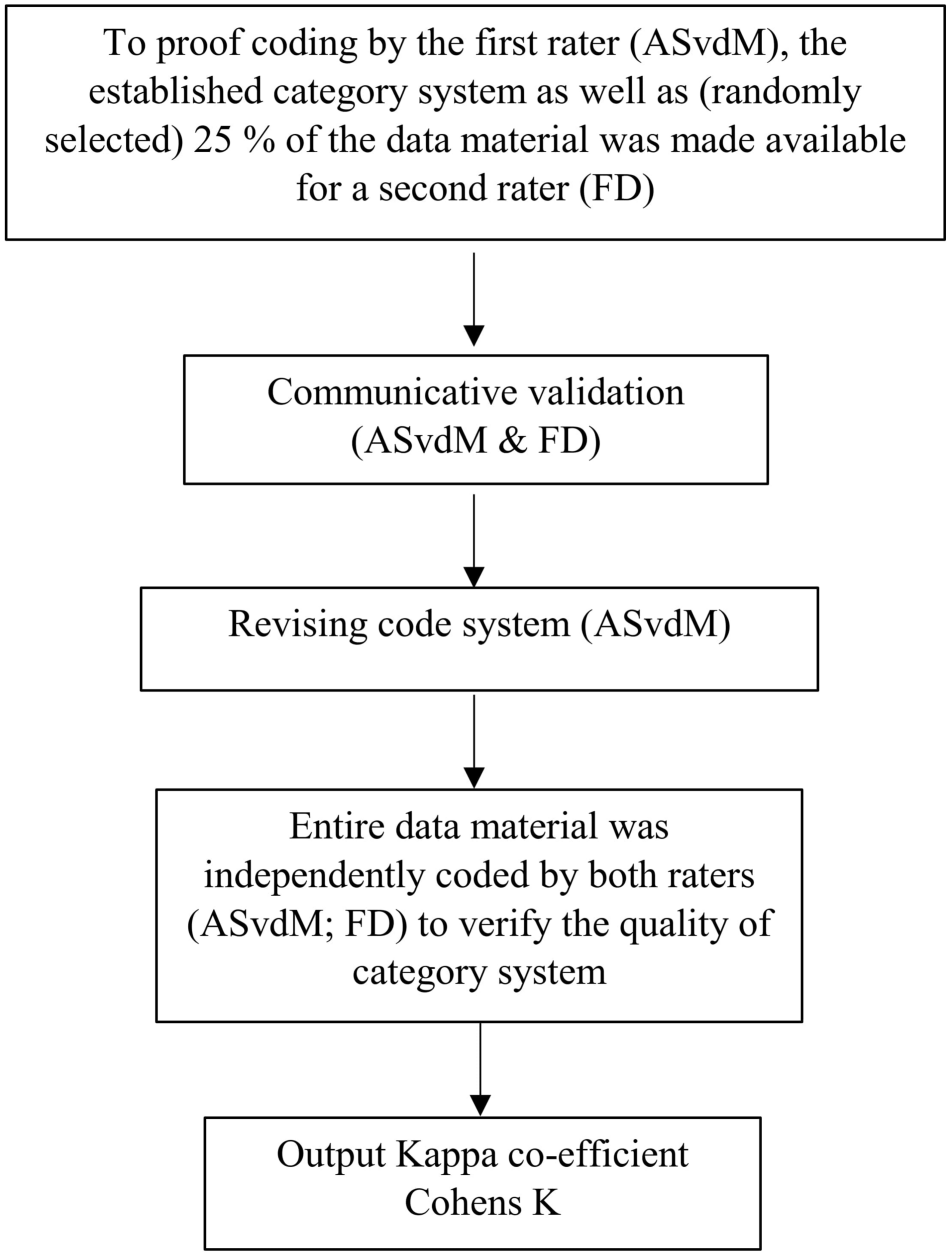
Calculation of inter-rater reliability.

In this study, the quality criteria we applied were rule guidance, procedural documentation, and interpretation assurance (Mayring, 2015). The analysis units were edited sequentially and systematically according to pre-defined rules. All analyses steps were documented in writing. Inter-rater reliability was assessed in two stages. To prove the first rater’s (ASvdM) coding, the established category system and (randomly selected) 25% of the data material was made available for the second rater (FD). After a communicated validation of the coding manual, the category system was revised and the entire data material independently coded by both raters (ASvdM; FD) to verify the quality of the category system. Inter-rater reliability was established with Kappa coefficients according to Cohen (κ) using the corresponding function in MAXQDA, 2020. An adjustment is made for the probability of random matches. A check was made at segment level to ensure that the codes match. Our aim was to achieve a code overlap of at least 60% at segment level. Inter-rater reliability was calculated separately for each group (S1; S2; S3). The Kappa coefficient calculation resulted in κ = 0.69 for interview material of S1, κ = 0.74 for interview material of S2 and κ = 0.62 of interview material of S3, indicating good inter-rater reliability (Greve and Wentura, 1997). The calculation of inter-rater reliability is shown in detail in Figure 2.

## Results

The main categories deductively formed along the structured guideline, as well as the subcategories inductively formed on the material, were assigned to these four topics:

(1) Conception of illness.
(2) Conception of health.
(3) Knowledge about health care structures in the country of origin.
(4) Perceptions regarding mental health care structures in Germany.

Twelve main categories in all were generated. The main categories are composed by the contents of the subcategories. Together with concise excerpts of the interviews, these are explained below.

### Conception of illness

Five main categories, abbreviated in the following headings as MC, were assigned to the topic *Conception of illness*. Detailed information on the configuration of the main and subcategories is presented in Table 2. Considering the number of codes of the main categories, it is noticeable that the main category *Somatic illness* has more codes (107) than the main category *Mental illness* (73). Likewise, more codes were assigned to the main category *Known somatic health interventions* (58) than to the main category *Known mental health interventions* (15).

**Table 2:** Generated categories for the topic conception of illness.

Main category	Subcategories	Codes total	Amount of documents	Findings in study population
Somatic illness	5	107	24	S1; S2; S3
	Described symptoms of somatic illness	59	24	S1; S2; S3
	Described mood during somatic illness	24	18	S1; S2; S3
	Appraisal of somatic illness	8	6	S1; S2; S3
	Unclear description of somatic illness	8	6	S1; S3
	Described causes of somatic illness	8	5	S2; S3
Mental illness	6	73	23	S1; S2; S3
	Described symptoms of mental illness	32	15	S1; S2; S3
	Appraisal	16	10	S1; S2; S3
	No knowledge	9	9	S2; S3
	Unclear description	8	7	S1; S2; S3
	Named causes	5	4	S1; S3
	Described mood	3	2	S3
Known somatic health interventions	7	58	23	S1; S2; S3
	Medical consultation	23	16	S1; S2; S3
	Medication	14	11	S1; S2; S3
	Self-care	9	7	S1; S2; S3
	Activity	4	3	S3
	Praying	4	2	S3
	Believing in recovery	3	2	S3
	No rumination	1	1	S1
Known mental health interventions	5	15	8	S1; S2; S3
	Psychotherapy	4	4	S1; S3
	Being active	4	2	S2; S3
	Conversation with friends or caregivers	2	1	S3
	Sports	2	2	S1
	Knowledge about mental illness	1	1	S1
	Self-care	1	1	S3
	Medical consultation	1	1	S1
Changed concept of illness	4	33	19	S1; S2; S3
	Increased HL	15	12	S1; S3
	No significant modification	9	7	S1; S2; S3
	Detachment from religious concepts of illness	5	3	S1; S3
	Now more often or more seriously ill	4	4	S1; S3

Coded document = one respondent; maximum of coded documents = 24.

#### MC: somatic illness

Our respondents described somatic-illness symptoms as having no appetite, being very tired, and having less energy. Furthermore, different types of pain, such as abdominal pain, headache or back pain were mentioned. Named diseases, apart from colds and coughs, were AIDS, Ebola, cancer, diabetes, and Covid-19. Respondents described mood and associated emotions during somatic illness as dissatisfaction, sadness, and tiredness. One respondent also reported worrying about dying through illness:

‘When I get sick and am afraid I’ll die, I pray.’ (S3_R9)

Respondents appraised somatic illness as something negative. One respondent (S3_R2) stated:

‘Yes, in my opinion, illness is like that - when you can't walk or whatever, when you're in a wheelchair or something, that's the worst, (...) for me of course. If you always, always need help and you always have to ask somebody, personally, I'd rather die than be in a situation like that. I think that's better.’

Eight of the respondents did not describe expressions of illness or potential manifestations. The respondents named smoking, drug and alcohol consumption, unhealthy food, lack of cleanliness, not enough sleep and bad weather as causes for getting a somatic disease.

#### MC: mental illness

The respondents described mental illness by naming depression, addiction, traumatization, severe anxiety, nightmares, soliloquies, lack of motivation, social withdrawal, fanatical beliefs or “forces of nature that have occupied the mind.” One respondent defined an abscess as an expression of mental illness. Another respondent defined cognitive disability as a mental disease. The term ‘sick in the head’ was also used by some respondents to describe mental illness.

S1_R6: ‘And if you are sick in your head, you do not notice it (laughs).

Interviewer: ‘You do not notice?’

S1_R6: ‘No, then the others notice (laughs).’

Nine respondents stated that they did not know what mental illness is. Eight respondents said that they knew that mental illness exists but could not describe it. Respondents named poor sleep, disputes, traumatic experiences, stress, experiences of fleeing and the absence of parents as potential causes of mental illness. Only two segments could be coded, where respondents described the mood during mental illness. One respondent (S3_R9) said:

‘But when you are mentally ill, you want to protect yourself, to survive somehow. Experience nothing, just survive.’

#### MC: known somatic health interventions

Respondents named medical treatment and medication as a known somatic health intervention. In the subcategory *self-care* segments were coded with answers of the respondents that included self-care strategies like avoiding stress, drinking a lot and staying in bed when you are ill. Furthermore, respondents named healthy nutrition, praying and to believe in your own recovery as a possible intervention for somatic illness.

#### MC: known mental health interventions

The interventions mentioned by the respondents were psychotherapy, social activities, sports, self-care, medical consultation and knowledge about mental illness. Respondents also named talking to friends or caregivers as a helpful strategy.

#### MC: changed concept of illness

Twelve respondents stated that their knowledge about disease and how to deal with it had improved since coming to Germany. On the one hand, they had learned more about the causes and ways in which diseases spread since their arrival. On the other hand, they had gained knowledge about disease prevention, compared to the time before coming to Germany. Three respondents stated that they did not know the difference between mental and somatic illnesses before coming to Germany.

‘(...) Before/when I was a child, I didn't exactly know what mental and somatic illness is. But in Germany, of course, it became clearer to me, and now as you're an adult, you discuss things more.’ (S3_R7)

One respondent reported that his own mental illness had increased his knowledge and dealing with it.

‘Well, I used to be like that, all childish. Okay, I had no idea what to do, whenever I got sick, I went to the doctor and stuff. Otherwise, not at all, well, I didn't know what to do when you get crazy, what to do when you are sick in the head, (...). But then I came here [Germany], I experienced it myself, and I learned what to do.’ (S1_R6)

Other respondents named that they have now a greater knowledge about health-promoting behaviors, such as drinking water, doing sports and paying attention to nutrition. The respondents also cited the transfer of knowledge by doctors, caregivers and school as the cause for their increase in knowledge and competence. Seven respondents said that their concept of illness had not changed in recent years and months. Three respondents said that their approach to illness and knowledge of illness had changed because they had distanced themselves from religious concepts of illness.

‘It used to be like that (...) I was also 13 years old at the time. Being ill was for me, so it had something to do with God a bit. That God can help me.’ (S3_R9)

Four respondents also mentioned that they now pay more attention to disease-related topics since their arrival in Germany because they are now more often ill. Causes named were more stress through work and school, and more infections or injuries through activities like playing soccer.

### Conception of health

Three main categories were assigned to the topic *Conception of health*. Detailed information on the configuration of the main and subcategories is presented in Table 3.

**Table 3:** Generated categories for the topic conception of health.

Main category	Subcategories	Codes total	Amount of documents	Findings in study population
Described aspects of health	6	90	24	S1; S2; S3
	Happiness	25	18	S1; S2; S3
	Healthy and active body	25	16	S1; S2; S3
	Health as a high value	22	20	S1; S2; S3
	Absence of illness, pain and problems	10	10	S1; S2; S3
	Healthy mind	6	4	S1; S3
	Unclear description of health	2	2	S2; S3
Personal health care strategies	10	82	22	S1; S2; S3
	Nutrition	30	19	S1; S2; S3
	Self-care	15	13	S1; S2; S3
	Sports	11	9	S1; S3
	Social activities	7	6	S1; S3
	Room cleanliness	4	4	S1; S3
	Positive thinking	4	4	S1; S2; S3
	Unclear description	4	3	S1; S2
	Body hygiene	3	3	S1; S3
	Moderate consumption of alcohol and nicotine	3	2	S3
	Sleep	1	1	S3
Modifications in concept of health	5	24	16	S1; S3
	Increased HL	10	8	S1; S3
	Changed attitude to life	4	4	S1; S3
	More focus on health	4	4	S1; S3
	Changed eating habits	3	3	S1; S3
	No noticed modification	3	3	S1; S3

Coded document = one respondent; maximum of coded documents = 24.

#### MC: described aspects of health

Respondents associated health with happiness, satisfaction, and gratitude. They said through health, one can take care of oneself and go to work. Furthermore, respondents defined health by having a healthy body and an active lifestyle, expressed in regular school attendance, doing sports and social activities. Health was also described as the absence of pain, illness, and problems. One respondent said that is it normal to have worries, but if they are not excessive, you are healthy. Four respondents described a healthy mind as a health aspect. They defined a healthy mind as possessing good ability to concentrate, have clear thoughts and being brave enough to go out without your friends.

#### MC: personal health care strategies

Most respondents highlighted the importance of healthy food for personal health care. One respondent (S3_R1) said:

‘Um, eating the wrong food, for example: If you cook something, if the window is open, then a lot of bacteria (.) gets on it, then you eat, then you don't see, then you get sick (..). Or the food sticks, I don't know, two three days outside, not in the refrigerator, if you eat then you get sick.’

The respondents mentioned sugary foods, strongly spiced foods or raw meat as unhealthy. One respondent said that it is unhealthy to eat ice cream or cold food in winter, as these would make you sick. Furthermore, aspects of self-care, like doing things that make you happy, living moderately, taking care of yourself, being outdoors and dressing according to the weather were mentioned as health-promoting behavior. Four respondents said it is important to think positively to stay healthy. Furthermore, good body hygiene and cleanness were mentioned as relevant for health care. Regarding alcohol and nicotine consumption, one respondent said that it is important to consume moderately and not exaggerate.

#### MC: changed concept of health

The respondents were asked if they noticed any modifications in their concepts of health compared to the time before fleeing to Germany. Eight respondents reported a higher level of knowledge, e.g., the necessity of regular medical appointments or which medical services are offered and accepted. Furthermore, they mentioned better accessibility to natural resources like water for regular body hygiene. Access to preventive medical check-ups were cited as a further gain in resources, too, bringing the issue of health maintenance into closer focus for the respondents. Some respondents stated that a change in their attitude toward life had also altered their perception of health. One respondent (S3_R9) said:

B: At the beginning I thought, heh, why are they acting so childish, even though they’re grown up having fun. With us, all adults are just adults. And here, when you see an adult caretaker having fun, I thought, hey, she's a woman, not a child, why she have fun? (...) she was actually healthy, I was sick then, maybe, I don't know. Maybe I'm still sick, but now it's completely changed. For me, anyone who has fun in life is healthy, anyone who takes life too seriously is not healthy for me.

I: And how did you get this attitude?

B: By seeing so many other people. For example, the adults who are having fun (...). There is no such thing that an adult must act like this, a doctor must act like that, with us it's highly structured, you know, children must act like that, a 15-year-old must act like that.

### Knowledge about health care structures in the country of origin

Three main categories were assigned to the topic *Knowledge about health care structures in the country of origin*. Detailed information on the configuration of the main and subcategories is presented in Table 4.

**Table 4:** Generated categories for the topic knowledge about health care structures in the country of origin.

Main category	Subcategories	Codes total	Amount of documents	Findings in study population
Knowledge about mental health care structures	8	62	23	S1; S2; S3
	No or rare offers	14	12	S1; S2; S3
	Inpatient psychiatric care	12	7	S1; S2; S3
	No knowledge about mental health care	8	6	S2: S3
	Family care	6	5	S1; S2; S3
	Religious care and habits	10	6	S1; S2; S3
	Outpatient medical care	5	4	S1; S2
	Spiritual ceremonies	4	3	S2; S3
	Psychotherapeutic care	3	3	S1; S3
Knowledge about somatic health care structures	4	11	6	S2; S3
	In- or outpatient medical care	6	5	S2; S3
	Spiritual ceremonies	2	2	S2; S3
	Religious care and habits	2	2	S3
	Family care	1	1	S3
Known concepts of illness	5	9	5	S1; S3
	Illness as a ‘work of god’	3	1	S3
	Illness as ‘problem with the head’	2	2	S1
	Illness ‘through ghosts’	2	1	S1
	Mental illness as ‘untreatable illness’	1	1	S1
	Illness as madness	1	1	S1

Coded document = one respondent; maximum of coded documents = 24.

#### MC: knowledge about mental health care structures

Most respondents stated, that there were no or rare offers for mental health care in their country of origin. Some respondents were unsure about the existence of care services and said that if they did exist, they were more likely to be in large cities. Some respondents talked about inpatient psychiatric care:

‘But I know something about the capital, because I read it on the internet, Damascus, Syria. Because if you have something in your brain or something like that, you are sent straight to a locked hospital. Then you go crazy. That's what they call crazy.’ (S1_R8)

Six respondents could not provide any information on mental-health care structures in their country of origin. Furthermore respondents stated that if someone is mentally ill, the family must care for that person. Religious practices like praying, reading the Quran, or contacts like the imam or a priest were named as mental-health care structures by six respondents as possibilities in case of mental illness. Also, spiritual ceremonies were reported by the respondents:

‘There is still this water, as I said, this healing water. If they are aggressive, you can let the [people] in there. They have to go in there twice a week to wash, but I don't know much about it.’ (S3_R9)

One respondent said that although such services exist, they are not used as often in Germany, because people are worried about basic supplies:

‘Because everywhere is, um, mental illness is everywhere is, but the problem is really a completely different one. In Germany, you have dinner, a roof over your head, food and money in your pocket at least as much as you need. Yes, in another country, the two countries I was allowed to experience, we didn’t think about psychology or whatever, because we had so many other serious problems (…).’ (S3_R7)

Psychotherapeutic care was named by three respondents as a known care structure for mental health in their country of origin.

#### MC: knowledge about somatic health care structures

Similar to the main category *mental health care structures*, inpatient and outpatient medical care, family care and healing through religious or spiritual acts were named as care structures by the respondents. Respondents emphasized the scarcity of medical care and its exorbitant costs.

#### MC: known concepts of illness

Five respondents reported being aware of mental-illness concepts from their country of origin that they do not share themselves. They stated that sometimes mental illness is seen as ‘work of God’, who determines the health or illness of people. According to other concepts, respondents stated that if someone is mentally ill, they must be possessed by a spirit and can only be treated by a healer. They also mentioned that mental illness is defined as a ‘problem of the head’ and that some mental illnesses are ‘untreatable’.

### Perceptions regarding mental health care structures in Germany

Only one main category was assigned to the topic *Perceptions regarding mental health care structures in Germany*. Detailed information on the configuration of the main category is presented in Table 5.

**Table 5:** Generated categories for the topic perceptions regarding mental health care structures in Germany.

Main category	Subcategories	Codes total	Amount of documents	Findings in study population
Understanding of psychotherapeutic care	5	39	18	S1; S3
	As biographical and solution-oriented conversations	27	15	S1; S3
	As support for problem solving	5	4	S1
	Unclear perception of psychotherapeutic care	4	3	S1; S3
	As prescription of medication	2	2	S1; S3
	As intelligence diagnostics testing	1	1	S3

Coded document = one respondent; maximum of coded documents = 24.

#### MC: understanding of psychotherapeutic care

Respondents of S1 and S3 mentioned in particular biographical and solution-oriented conversations as a part of psychotherapeutic care. One respondent said:

‘That you [psychotherapist] guide me like a little child and guide me back into life and I get along again. And those topics I never wanted to talk about with normal people/You are like mirror for me, for these things where my compatriots look at me so strangely, you are always open. Then you're a good mirror.’ (S3_R9)

Furthermore, four respondents also named support for problems as a psychotherapeutic scope. Three respondents could not describe aspects of psychotherapeutic care.

‘Therapist? I don't know, I didn't study. (..). That's different, that's not like family doctor (...). Therapist? That’s a much different thing. I don't know that.’ (S3_R5)

### Coding comparison among the samples

After presenting our total sample’s results along the main categories and their subcategories, sample differences are presented below alongside the comparison of the subcategories’ code segment sizes. This analysis of coding at the sample level enables an exploratory comparison of participants considering age differences, effects due to the different types of housing and clinical to non-clinical problems. The main categories *mental illness*, *personal health care strategies* and *known mental health interventions* were selected for further analysis, based on their relevance for psychotherapeutic practice, research and MHL.

#### Comparing sizes of code segments in subcategories of the main category mental illness

**Figure 3:**
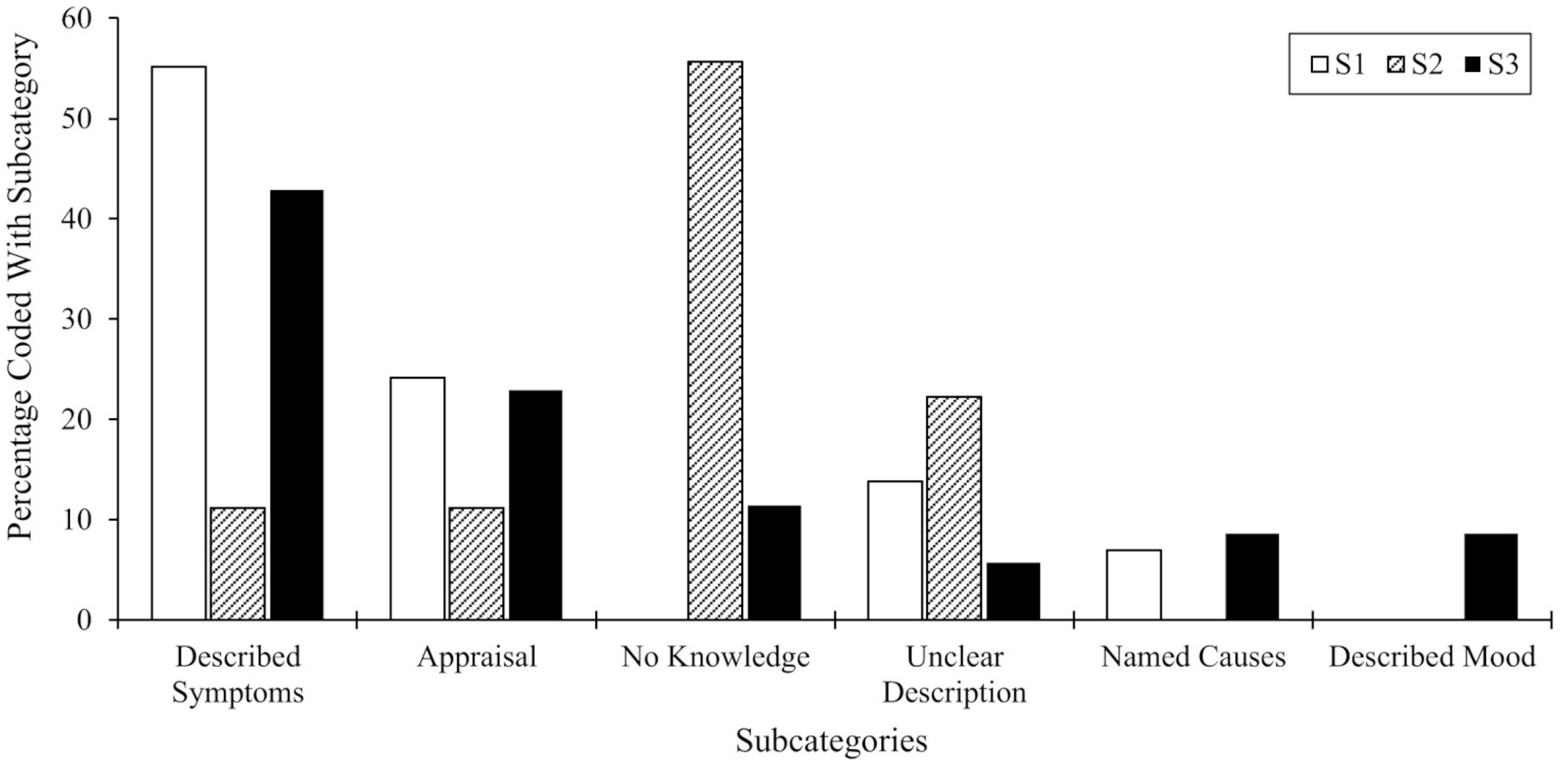
Comparison of code segment size of subcategories of the main category mental illness.

Considering the coded segment sizes of the subcategory *Described symptoms*, we observed more coding in the interview material of S1 (55.2%) and S3 (42.9%), i.e., adolescent participants, than in the younger S2 material (11.1%). Compared to the interview material of S1 (24.1%) and S3 (22.9%), fewer coding of the subcategory *Appraisal* (of mental illness) could be made in the material of S2. Furthermore, we coded the *No knowledge* subcategory with a segment size covering 55.6% in S2. 11.4% of coded segments were assigned to the subcategory *No knowledge* also in S3’s data. In comparison, no coding was undertaken in S1’s data, which discloses knowledge about mental illness in the group with clinical problems. The highest percentage of code segments regarding the *Unclear description* subcategory was also made in S2’s interview material (22.9%), a finding in line with our analysis’ previous outcomes showing that younger respondents could not at all or just barely define mental illness or aspects thereof. In the older S1 and S3 samples, we coded segments for this subcategory, thus indicating some concepts of mental illness among some respondents. S2’s data enabled no coding for the *Named Causes* subcategories; however, this subcategory yielded a similar amount of code-able material for S1 and S3, namely with 6.9% in S1 and 8.6% in S3. Detailed information on our comparison of code segment size of subcategories of the main category mental illness is found in Figure 3.

#### Comparing sizes of code segments in subcategories of the main category personal health care strategies

**Figure 4:**
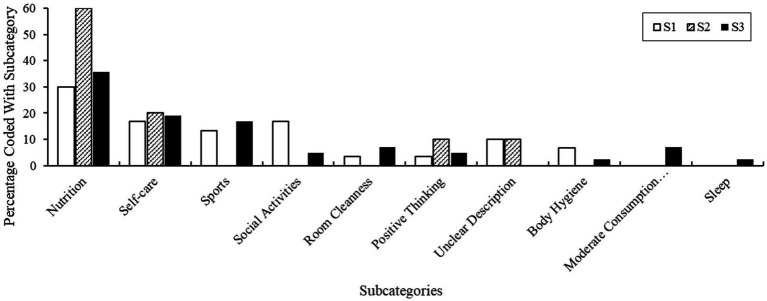
Comparison of code segment size of subcategories of the main category personal health care strategies.

The coded segments of the main category *Personal health care strategies* were almost equal in size. The exception here was subcategory *Nutrition’s* segment coding in S2 material. Compared to the material from S1 (30%) and S3 (35.7%), we found that a total of 60% coded segments were retrievable from S2 material that were assignable to this subcategory. In the material from all samples, we coded similarly sized segments assigned to the *Self-care* subcategory. Relatively medium-sized segments, were found in the material of S1 (13.3%) and S3 (16.7%) for the subcategory *Sports* and in the material of S1 for the subcategory *Social activities* with a segment coverage of 16.7%. Again, we detected fewer coded segments across subcategories in S2 material than in S1 and S3 material. S1 and S3 participants’ responses to health promotion strategies were similar, evident in their segment coverage across subcategories. S1 material only enabled more codes regarding the *Social Activities*, *Body Hygiene* and *Unclear Description* subcategories. Our illustration of the comparison of coded segment sizes can be seen in Figure 4.

#### Comparing sizes of code segments in subcategories of the main category known mental health intervention

**Figure 5:**
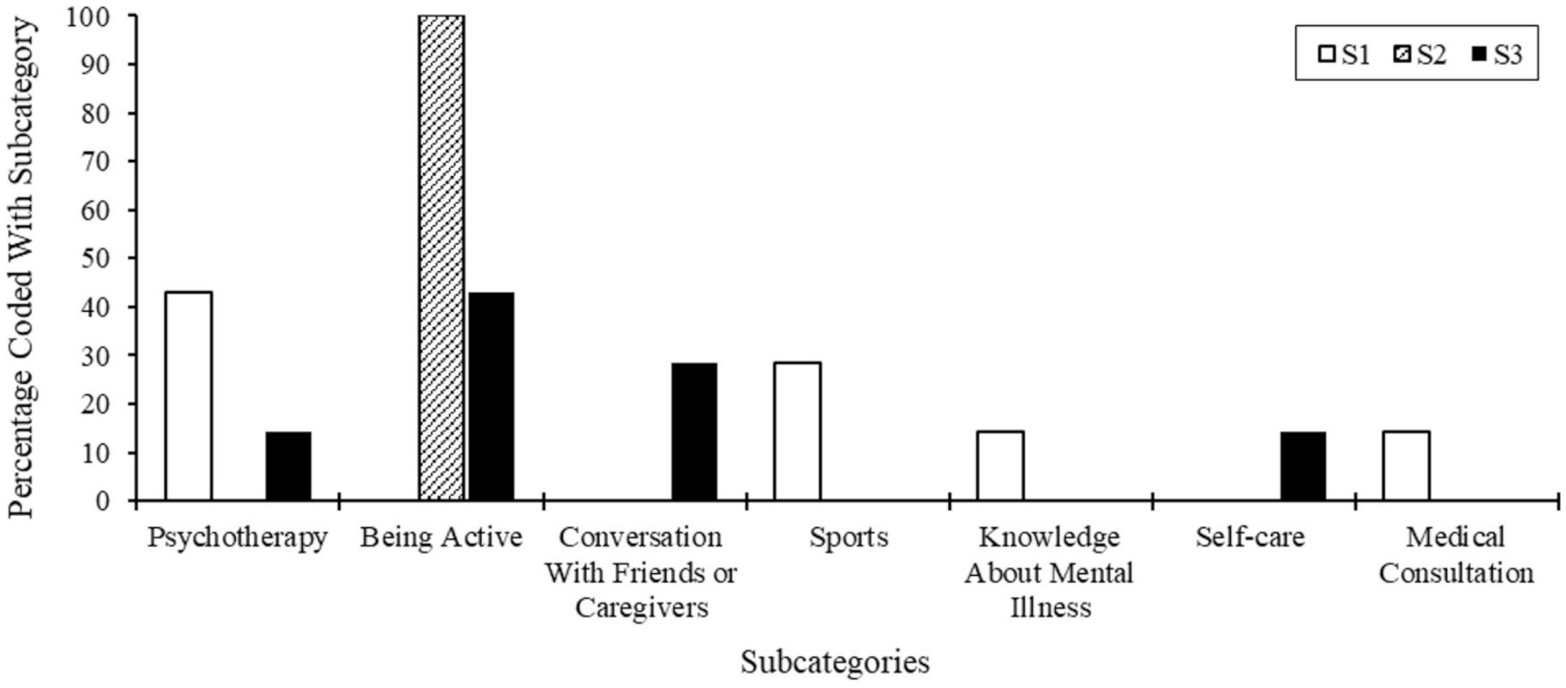
Comparison of code segment size of subcategories of the main category known mental health intervention.

After comparing the segment sizes of subcategories of the main category *Known mental health interventions*, we found that all segments in S2 interview material were coded in the *Being active* subcategory. The remaining interview material from S2 yielded no coded segments. In S1’s interview material the *Psychotherapy* subcategory was the one with the most frequently coded interview material (42.9%), followed by *Sports* (28.6%) and *Medical Consultation* (14.3%) in terms of segment size. When considering the S3 interview material’s coded segment sizes, the *Being Active* subcategory also proved to be the subcategory with the largest coded area (42.9%), as was the case in S2’s interview material. In this analysis of the main category, our sample comparison shows that the younger S2 participants were least able to provide information on known mental health interventions. Compared to S1, S3 participants mentioned social and active mental health interventions (*Being active*; *Conversations with friends and caregivers*). Our diagram of the comparison of coded segment sizes is in Figure 5.

## Discussion

To assess conceptions of illness and health in refugee youths, we conducted a semi-structured interview. To classify our results, we examined their knowledge on both mental as well as somatic illness and health. Below we discuss our study results in the context of prior research, and implications for psychotherapeutic practice, research and MHL.

### Differences in the extent and quality of knowledge

The subcategories of the main category *Somatic illness* were coded more frequently than the subcategories *Mental illness* while analyzing our interview material. Due to this we conclude, that the extent of knowledge about somatic illnesses is greater than that of mental illnesses in refugee youth. However, the interview excerpts suggest some misconceptions about potential health hazards, e.g., for example, that eating ice cream leads to a cold. Little knowledge and difficulties in understanding the term *mental health* of refugee youth are factors already reported in previous studies where Syrian refugee adolescents were interviewed (Filler et al., 2021). This is also reflected by the fact our respondents revealed next to no knowledge about or any awareness of psychotherapy in Germany. Based on this result we suggest to impart basic, age-appropriate knowledge about illness concepts at the beginning of therapy to facilitate disorder-specific psychoeducation. However, individual and religious concepts of health and illness should also be taken into account, to prevent them from becoming barriers in psychotherapeutic work (Ellis et al., 2010; Colucci et al., 2015).

A mandatory questionnaire introduced at the beginning of therapeutic contact to capture personal (mental) health-related concepts of children and adolescents would help to meet this need. Concerning knowledge about health care treatments, our respondents knew more about treatment options for somatic than for mental health. One potential reason for this is the higher utilization of somatic health care services and comparatively low utilization of mental health care services by refugee youth (Barghadouch et al., 2016). Furthermore, in our study respondents that are living in YWF possessed more knowledge about health care structures, than respondents living with their families, stating that they had been introduced to the German health care system by staff at their YWF and were thus in contact with various health care services. To counteract the barriers identified in our and previous studies concerning the utilization of mental health services in particular, detailed information should be made available to refugee families regarding local health structures. Information should include details regarding the accessibility of specific health care offer, the kind of offer (outpatient or inpatient) and the scope of the offer (which health sector is addressed?). Therefore, future research should focus on the analysis of (M)HL of families in order to create offers that meet the needs of families. Research on the transgenerational transmission of health-related knowledge in refugee families, before and after flight, would also be relevant to capture external factors that threaten or promote the transmission of MHL.

### Differences in appraising somatic and mental illness

Our study’s respondents revealed more negative attitudes toward mental illnesses than toward somatic illnesses. This finding of ours is also in line with previous research in which UASC in particular were interviewed (Byrow et al., 2020). Our participants’ impressions of psychiatric admissions in their country of origin were mainly negative. Negative beliefs and assumptions about mental illness and care (like fearing stigmatization) can impede further engagement with mental health issues (Majumder et al., 2015; Aguirre Velasco et al., 2020). Young people may avoid attending informational or educational services on mental health topics, thus preventing any improvement in MHL, which in turn would hinder the utilization of mental health care among young refugees (Filler et al., 2021). To counter this, refugee children and young people in particular should have access to low-threshold information services to be accessed without the help of third parties such as interpreters. Previous studies have shown that working with interpreters can discourage young refugees from speaking openly about their mental health issues if they fear the interpreter might know someone in the refugee’s family or if they differ greatly in age, dialect, or ethnicity (Colucci et al., 2015).

Such services could be information websites or apps configured in different languages not requiring that users provide personal data. Another possible way to promote MHL while addressing stigma concerns might be the implementation of health-related knowledge in school curricula. This might make it easier for (refugee) students to engage with and learn about mental health content within their peer group. In psychotherapeutic settings, data protection should be considered and sufficiently explained to refugee patients. All persons involved in therapy, e.g., youth welfare workers, should also be informed in this regard and be asked to respect confidentiality.

### Factors promoting MHL

The respondents describe health as an important value, which goes hand in hand with an active and healthy lifestyle and body. Given the importance of health, the associated feeling of happiness, and taking into account that attitudes regarding mental health facilitate recognizing and seeking help (Jorm, 2012), this can be seen as a MHL promoting factor. Also, the amount of known health promoting strategies described by the interviewed children, adolescents and young adults demonstrates that they have knowledge of health-promoting behaviors, however mostly regarding somatic health. One exception were social activities as a mental health-promoting strategy, which follows research findings that have identified social contact as a protective factor with respect to mental illness (Höhne et al., 2020).

Despite language barriers, psychological stress, and living alone and away from their families, it was our older S1 andS3 respondents who demonstrated more knowledge about health and illnesses. Further research should therefore explore the MHL of refugee children and adolescents growing up in families longitudinally to examine the influence of age, length of residence, and family factors on MHL. Our research suggests that growing up in YWF might be an MHL-promoting factor for some of the respondents, as the knowledge of staff and compulsory involvement with the healthcare system leads to knowledge regarding the use of healthcare services. On the other hand, considering that the majority of refugee youths placed in youth welfare in Germany are unaccompanied refugees and live in Germany without close relatives, it is possible that fleeing unaccompanied as well as the unaccompanied stay of the youth may be associated with greater independence, and a greater need of self-care, which may positively influence MHL. Future research should therefore investigate whether unaccompanied status is a factor promoting MHL. There is evidence of high psychological stress among refugee children and adolescents (Blackmore et al., 2020). As we expected, our study indicated that patient sample S1 participants had more knowledge about mental illness and its treatment options than the S2 and S3 respondents with no reported mental burden, as they were already involved in the diagnostic process at a psychotherapeutic outpatient clinic when they were interviewed. However, this result not necessarily implies that MHL is related to psychological stress, since a number of confounding factors (e.g., selection bias) might influence this association. Again, longitudinal studies could shed light on whether and how mental illness influences MHL in refugee children and adolescents.

## Conclusion

Our research revealed that refugee youth show more knowledge regarding somatic than mental illness and their respective treatment. Our findings reveal the need to improve the MHL of refugee children, adolescents and young adults, since low MHL can lead to low utilization of mental health services and associated chronicity of disorders. To help alleviate the known stigmatization fears of respondents, low-threshold services would be a means through which such populations could gain knowledge about mental illnesses and their treatments. Finally, our study also revealed MHL-facilitating factors such as living in YWF. To verify these results, longitudinal studies that also compare refugee and non-refugee populations should be pursued.

### Study limitations

A limitation of our study is a missing comparison group of non-refugee youths. This limits the interpretation of our results in the sense that we have no norm values to compare the responses of our participants to. As this is a major issue for many refugee-related MHL projects, we are currently conducting a qualitative study of MHL in German adolescents (Durlach et al., in preparation)^1^. An additional limitation is our sample’s composition. We included more male adolescents and young adults than children or female adolescents or young adults in our research. Also, the majority of respondents were unaccompanied refugees. Recruitment of participating youth and young adults stopped when content saturation was reached. Unfortunately, due to the outbreak of the COVID-19 pandemic was not possible to recruit further refugee children at schools, even though a larger number of participants would have been desirable here. On the one hand, the heterogeneity of the sample in total and in terms of the broad age range and the diversity of settings from which the participants, children, adolescents, and young adults, were recruited, can be seen as a strength of the study. On the other hand, this heterogeneity of our groups also limits conclusions regarding group differences. Further studies should, as already mentioned, target refugee families and especially female children and adolescents. Furthermore, when considering the results, it must be taken into account that only patients who feel comfortable in the outpatient therapeutic setting agreed to take part in the study. Therefore, it cannot be ruled out that their response behavior was influenced by a basically positive attitude toward health care. As some of these interviews were conducted with help from interpreters, we cannot rule out that some statements from the interviewees were incompletely or incorrectly conveyed. The translation of the interview material from German into English may also have led to data imprecision. To counter this, we conducted our analysis on the German material and only translated it into English afterwards. The aspect of potential data loss due to inaccurate translations should be considered in future qualitative interview designs involving multilingual samples when conceiving the research design.

## Data availability statement

The raw data supporting the conclusions of this article will be made available by the authors, without undue reservation.

## Ethics statement

The studies involving human participants were reviewed and approved by Ethics Committee of the Department of Psychology of Philipps University Marburg. Written informed consent to participate in this study was provided by the participants’ legal guardian/next of kin.

## Author contributions

AvdM, FD, and HC were responsible for the conception and design of work. AvdM was responsible for conducting the interviews, data collection, and the initial qualitative data analysis, and responsible for drafting the article. AvdM, KS, and FD were involved in the data interpretation. HC was responsible for supervision of the research study and the article. KS, FD, and HC provided critical feedback and helped shape the research, analysis, and manuscript and gave final approval of the version to be published.

## Funding

Open Access funding provided by the Open Acess Publishing Fund of Philipps-Universität Marburg with support of the Deutsche Forschungsgemeinschaft (DFG, German Research Foundation).

## Conflict of interest

The authors declare that the research was conducted in the absence of any commercial or financial relationships that could be construed as a potential conflict of interest.

## Publisher’s note

All claims expressed in this article are solely those of the authors and do not necessarily represent those of their affiliated organizations, or those of the publisher, the editors and the reviewers. Any product that may be evaluated in this article, or claim that may be made by its manufacturer, is not guaranteed or endorsed by the publisher.

## Footnotes

^1^Durlach, F., van der Meer, A.S., Stracke, M., and Christiansen, H. (in preparation). "I don’t know what it means, because otherwise I would do it better." Mental health literacy of children and adolescents with mental illnesses. A qualitative content analysis.
