# Supplementary material for: “I can’t describe how I could get better, but I would like to” - Conception of health and illness of refugee youth in Germany
Source: Front Psychol. 2023 May 12;14:1107889. doi: 10.3389/fpsyg.2023.1107889 (PMC10213462; doi:10.3389/fpsyg.2023.1107889)
Supplement: Supplementary file 1 [file Table_1.DOCX]

**Topic 1: Conception of illness**

1. What does it mean to be ill?
2. What does being ill mean to you?
3. How do you notice when you are ill? What is different then?
4. Is being sick something physical?
5. What can you do about being ill?
6. Has your concept of being ill changed?

**Topic 2: Conception of health**

1. What does it mean to be healthy?
2. What does being healthy mean to you?
3. How do you stay healthy?
4. Do you have any influence on staying healthy?
5. Is it important for you to be healthy?
6. Has your idea of being healthy changed?

**Topic 3: Care**

Introduction: “We work in a child and adolescent psychotherapeutic outpatient clinic. We work with children, adolescents and young adults who may have many worries, many fears or are often very sad, meaning, they are mentally ill.”

1. Do you know similar mental health care services in your home country?
2. How do you think psychotherapy works? *(only S1 sample)*
3. What do you think a psychotherapist does? What’s the job of a psychotherapist? *(only S1 sample)*
